# Supplementary material for: The Maternal Diet with Fish Oil Might Decrease the Oxidative Stress and Inflammatory Response in Sows, but Increase the Susceptibility to Inflammatory Stimulation in their Offspring
Source: Animals (Basel). 2020 Aug 19;10(9):1455. doi: 10.3390/ani10091455 (PMC7552684; doi:10.3390/ani10091455)
Supplement: Supplementary file 1 [file animals-10-01455-s001.pdf]

Supplementary Table 1. Fatty acid composition of soybean oil and fish oil in experimental diets

Item	Fish oil	Soybean oil
Fatty acids(g/100g total fatty acids)		
14:0	7.59	n.d1
16:0	17.87	10.57
16:1	9.98	n.d
18:0	3.54	3.79
18:1	12.52	23.76
18:2(n-6)	1.19	53.89
18:3(n-3)	0.80	8.00
20:0	3.15	n.d
20:1	1.72	n.d
20:5(n-3)	21.12	n.d
22: 1	n.d1	n.d
22:5(n-3)	2.28	n.d
22:6(n-3)	14.18	n.d
n-6:n-3	0.03	6.70

n.d=not detectable; n-3 = n-3 poly unsaturated fatty acid; n-6 = n-6 ploy unsaturated fatty acid; n-6: n-3=n-6 polyunsaturated fatty acids: n-3 polyunsaturated fatty acids.

Supplementary Table2. Primers for all target genes used for real-time PCR

Genes	Primer sequence (5’–3’)	Product size(bp)	GenBank accession	Reference
β-actin	F: 5'-TCATCACCATCGGCAACGAG-3'	217	DQ845171.1	
	R: 5'-GCCGTGATCTCCTTCTGCAT-3'			
SOD	F:5'-GAGACCTGGGCAATGTGACT-3'	189	GU944822.1	[1]
	R:5'-CTGCCCAAGTCATCTGGTTT-3'			
GPx	F:5'-GCTCGGTGTATGCCTTCTCT-3'	103	NM_214201.1	[1]
	R:5'-AGCGACGCTACGTTCTCAAT-3'			
CAT	F:5'-ACTTCTGGAGCCTACGTCCT-3'	93	NM_214301.2	[1]
	R:5'-ATCCGTTCATGTGCCTGTGT-3'			
IL-1β	F:5'-TCTGCCCTGTACCCCAACTG-3'	64	NM214055.1	[2]
	R:5'-CCAGGAAGACGGGCTTTTG-3'			
IL-6	F:5'-ATCAGGAGACCTGCTTGATG-3'	177	NM_214399	[3]
	R:5'-TGGTGGCTTTGTCTGGATTC-3'			
TNF-α	F:5'-CCAATGGCAGAGTGGGTATG-3'	116	NM_214022	[3]
	R:5'-TGAAGAGGACCTGGGAGTAG-3'			
IL-10	F:5'-GGTTGCCAAGCCTTGTCAG-3'	202	NM_214041	[3]
	R:5'-AGGCACTCTTCACCTCCTC-3'			
TAB1	F:5'-CAGAGTTTGCCAAGCAGACC-3'	175	NM_001244067.1	
	R:5'-CTCAGCTCGCCCAGAGGATA-3'			
TAK1	F:5'-GGCTGTTCATAACGGTACTC-3'	204	KU504629.1	
	R:5'-TGGCCTTCATCTGAATACTG-3'			
PTGS2	F:5'-ATGAACGGCTGTTCCAGACG-3'	218	NM_214321.1	
	R:5'-AATCTGGAAGGCGTCAGGCA-3'			
ALOX5	F: 5'-GACCCCTGCACTCTGCAGTT-3'	201	XM_021072736.1	
	R:5'-GGTCTGGTGGACGTGGAAGT-3'			
GPR120	F:5'-CAGATCACCAAGGCATCAAG-3'	206	HQ662564.1	
	R:5'-GGCCAGATGACCAGGTTTTG-3'			
TLR-4	F:5'-TGTGCGTGTGAACACCAGAC-3'	136	NM_001113039	[3]
	R:5'-AGGTGGCGTTCCTGAAACTC-3'			
NF-κB	F:5'-TGCTGGACCCAAGGACATG-3'	60	AK348766.1	[2]
	R:5'-CTCCCTTCTGCAACAACACGTA-3'			
PPARγ	F: 5'-GTGCGATCTTAACTGTCGGA-3'	192	NM214379.1	
	R:5'-AGGTCAGCAGACTCTGGGTT-3'			

SOD superoxide dismutase, GPx glutathione peroxidase, CAT catalase, IL-1β Interleukin-1β, IL-6 Interleukin-6, TNF-α Tumor necrosis factor α, IL-10 Interleukin-10, TAB1 TAK1 binding protein 1, TAK1 Transforming growth factor-β activated kinase 1, PTGS2 Prostaglandin-endoperoxide synthase 2, ALOX5 lipoxygenase enzyme5, GPR120 G-protein coupled receptor 120, TLR4 Toll-like receptor 4, NF-κB Nuclear factor-B, PPARγ Peroxisome proliferator activated receptor gamma

Reference

[1] Su, G., J. Zhao, G. Luo, et al., "Effects of oil quality and antioxidant supplementation on sow performance, milk composition and oxidative status in serum and placenta," *Lipids Health Dis,*

vol. 16, no.1, pp. 107. 2017.

[2] Han, F., L. Hu, Y. Xuan, et al., "Effects of high nutrient intake on the growth performance, intestinal morphology and immune function of neonatal intra-uterine growth-retarded pigs," *The British journal of nutrition*, vol. 110, no.10, pp. 1819-1827. 2013.

[3] Pasternak, J.A., V.I.A. Aiyer, G. Hamonic, et al., "Molecular and Physiological Effects on the Small Intestine of Weaner Pigs Following Feeding with Deoxynivalenol-Contaminated Feed," *Toxins (Basel)*, vol. 10, no.1. 2018.
